# Alginate- and Gelatin-Coated Apple Pieces as Carriers for *Bifidobacterium animalis* subsp. *lactis* DSM 10140

**DOI:** 10.3389/fmicb.2020.566596

**Published:** 2020-10-16

**Authors:** Daniela Campaniello, Antonio Bevilacqua, Barbara Speranza, Milena Sinigaglia, Maria Rosaria Corbo

**Affiliations:** Department of the Science of Agriculture, Food and Environment, University of Foggia, Foggia, Italy

**Keywords:** apples, edible coatings, probiotics, alginate, carriers, gelatin

## Abstract

Fruit and vegetables are considered good natural supports for microorganisms; however, probiotics could cause negative changes on some organoleptic and sensory traits. Thus, the main topic of this paper was the design of coated apple chips as carriers for probiotics with a high level of sensory traits. The research was divided into two steps. First, four functional strains (*Limosilactobacillus reuteri* DSM 20016, *Bifidobacterium animalis* subsp. *lactis* DSM 10140, and *Lactiplantibacillus plantarum* c16 and c19) were immobilized on apple pieces through dipping of fruit chips in probiotic suspensions for different contact times (from 15 to 30 min) and stored at 4°C for 12 days. Periodically, the viable count was assessed. As a result of this step, a contact time of 15 min was chosen because it assured an optimal deposition of microorganisms. In the second step, apple pieces inoculated with *B. animalis* subsp. *lactis* DSM 10140 were coated with alginate and gelatin and stored at 4 and 8°C for 10 days; pH, microbiological counts, color (browning index), and sensory scores were evaluated. *Bifidobacterium animalis* DSM 10140 exerted a negative effect on apple chips and cause a significant browning; however, the use of coating counteracted this phenomenon. In fact, coated chips showed higher sensory scores and lower browning index. In addition, gelatin showed better performances in terms of probiotic viability, because at 8°C, a significant viability loss of *B. animalis* DSM 10140 (1.2 log cfu/g) was found on alginate-coated chips. Gelatin-coated apple pieces with *B. animalis* subsp. *lactis* DSM 10140 could be an attractive functional food for a wide audience, although further investigations are required on *in vivo* effects of this product after consumption.

## Introduction

Cell immobilization is the physical confinement of intact cells to a well-defined region of space; a requisite of immobilization is that cells retain their biological activity (Karel et al., 1985). This technology was successfully applied on food-grade microorganisms for technological purposes; for example, Moreno-García et al. (2018) reported that it is possible to confine intact and active yeasts to a specific region, increasing cell density, enhancing some metabolic pathways, improving the control and stability of the strain, providing cell protection against shear forces, and enabling cell recovery/reutilization.

Microorganisms (yeasts or bacteria) could be immobilized on various gels (polyacrylamide, alginate, pectinates, carrageenan, etc.) either of industrial origin (porous fibers, polyurethane foams, membranes, etc.) or natural materials (Poreda et al., 2013).

Fruit and vegetables are considered good natural supports for microorganisms, because of some positive properties, like the presence of ridges on the plant surface micro-architecture, the content in prebiotics, which protect probiotic microorganisms from the acidic environment of the stomach, and other nutrients (carbohydrates, vitamins etc.). Fruits are composed of non-digestible carbohydrates, which are the base for cell immobilization; due to their cellulose content, some fruits, such as apples and pears, may exert a protective effect on the probiotic microorganisms during passage through the intestinal tract allowing these microorganisms (such as *Lacticaseibacillus casei*) to reach the colon and benefit the host (Kourkoutas et al., 2006).

Fruit pieces are natural carriers considered very interesting to enhance the aroma characteristics of many products thanks to their abundance, low cost, and food-grade composition (Kandylis et al., 2012). Apple and quince pieces proved to be appropriate carriers for immobilization of *L. casei* cells (Kourkoutas et al., 2006), *Lactiplantibacillus plantarum* (Speranza et al., 2018), and *Saccharomyces cerevisiae* (Altieri et al., 2019).

On the other hand, modern consumers, increasingly attentive toward their health, have shown a growing interest in novel foods that are capable of preventing and/or curing illness (Mitropoulou et al., 2013). These healthy foods or “functional foods” exert beneficial effects, in addition to the traditional nutritional functions: they contain bioactive compounds, like dietary fiber, oligosaccharides, and active “friendly” bacteria as probiotics; some researchers reported that probiotic foods represent ca. 60–70% of the functional foods (Thakur et al., 2015). For a long time, probiotics foods have been a part of dairy products; however, some leading factors, like the increasing number of lactose-intolerant individuals or with allergy to milk proteins, as well as the emerging trend of vegetarianism, contributed to a renewed interest on not-dairy probiotic foods (mainly fruits and vegetables) (Valero-Cases et al., 2020).

The main aim of this paper was to design a carrier for functional microorganisms with apple pieces. This study was divided into different steps as follows: (i) adsorption of four functional strains (*Limosilactobacillus reuteri* DSM 20016, *Bifidobacterium animalis* subsp. *lactis* DSM 10140, and two *L. plantarum* strains) on apple pieces, focusing on the evaluation of strain survival and on the duration of the contact probiotic solution/fruits to design performing food-like systems; (ii) evaluation of the performances of *B. animalis* subsp. *lactis* DSM 10140 immobilized on apple pieces surrounded by two different coatings (alginate and gelatin) and stored at 4 and 8°C for 10 days, with a focus on the viability of the strain, sensory scores, and color of apple chips.

## Materials and Methods

### Strains

*Limosilactobacillus reuteri* DSM 20016 (Deutsche Sammlung von Mikroorganismen), *Bifidobacterium animalis* subsp. *lactis* DSM 10140, and *Lactiplantibacillus plantarum* c16 and c19 (Bevilacqua et al., 2010) were at −20°C in MRS broth (Oxoid, Milan, Italy) + 33% sterile glycerol (J.T. Baker, Milan, Italy).

Before each experiment, the strains were cultured in MRS broth (*L. reuteri* DSM 20016 and *L. plantarum* c16 and c19 at 30°C for 48 h and *B*. *animalis* subsp. *lactis* DSM 10140 at 37°C for 48 h under anaerobic conditions). Then, the cultures were centrifuged at 1200 × *g* for 10 min and the pellet was washed with sterile distilled water.

### Immobilization on Apple Pieces

Dipping solutions for the different treatments are reported in Table 1. The adsorption of the strains on apple pieces was achieved by a modified version of the method reported by Kopsahelis et al. (2007); peeled apple pieces of Granny Smith variety, approximately 2 × 2 cm (4.5 g), were used as support materials. Before immobilization, they were dipped into anti-browning (AB) solution, in order to prevent browning. For each strain, apple pieces (20–30 pieces) were introduced in 250 ml of cell suspensions (10^7^–10^8^ cfu/ml) for different times (15, 20, 25, and 30 min) (Altieri et al., 2019). After the dipping, the pieces were removed from the solution and stored into sterile containers at 4°C for 12 days. Apple pieces without microorganisms represented the control.

**TABLE 1:** Dipping solutions.

**Dipping solutions**	**Composition**
Anti-browning (AB)	Citrate 0.2% (w/v), ascorbate 1% (w/v), sterile water
Gelatin	Sucrose 0.5% (w/v), corn starch 0.08% (w/v), lemon juice 0.05% (v/v), distilled water
Alginate (A)	Alginate powder 2% (w/v), melted into sterile distilled water at 80°C
CaCl_2_ (hardening)	CaCl_2_ 0.5% (w/v)

### Evaluation of the Performances of *B. animalis* Subsp. *lactis* DSM 10140 Immobilized on the Apple Pieces With Alginate and Gelatin Coating

For gelatin coating, apple pieces were treated as follows: (i) dipping in AB solution, (ii) dipping in probiotic suspension for 15 min, (iii) dipping in gelatin solution, (iv) air drying for 10 min, and (v) storage at 4 and 8°C.

For alginate coating, samples were prepared by a modified version reported by Speranza et al. (2018). Apple pieces were first dipped into AB solution and then in alginate solution containing the probiotic (10^8^ cfu/ml) for 15 min; then, a dipping in CaCl_2_ for 5 min was done, followed by air drying for 10 min and storage in sterile boxes at 4 and 8°C.

Apple pieces with alginate or gelatin coating but without bifidobacteria were used as controls.

### Microbiological Analyses

Twenty five grams of apples were diluted into a sterile saline solution (0.9% NaCl) to obtain a 10-fold dilution and homogenized through a laboratory blender. Then, the homogenates were serially diluted, and the viable count was evaluated through the spread plate method on MRS agar; Petri dishes were incubated under anaerobic conditions (Anaerogen kit, Oxoid) at 30°C for *L. reuteri* DSM 20016 and *L. plantarum* c16 and c19 and 37°C for *Bifidobacterium* strain for 48 h.

### Colorimetric Analysis

Color was measured using a tristimulus colorimeter CR-300 Minolta Chromameter-2 Reflectance (Minolta, Japan); the measurement was performed on 10 different pieces of apple per sample. The calibration of colorimeter was done on a standard white plate (L^∗^ = 97.03, a^∗^ = + 0.01, and b^∗^ = + 1.63). Data were expressed according to CIELAB scale: L (luminescence), a (red/green coordinate), and b (yellow/blue coordinate).

### pH

pH was measured on apple homogenate (10 g of apple in 90 ml of saline solution) by a pH meter Crison, model micro pH 2001 (Crison, Barcelona, Spain); the calibration of the pH meter was done with standard buffer solutions at pH 2.0, 4.0, and 7.0.

### Sensory Assessment

The samples were evaluated to assess the sensory scores in terms of texture, odor, and overall acceptability. The panel consisted of 10 panelists, students, and researchers of the Department of the Science of Agriculture, Food and Environment. The samples were coded by a letter and presented individually in a random order to each panelist. The score was assigned using a scale ranging from 0 to 5 (where 0 = very poor, 2 = acceptability limit, 5 = excellent).

### Statistics

The experiments were performed on two independent batches and repeated twice for each batch. The results for the viable count on the second step were also modeled as decrease/increase compared to the inoculation (Δ*C*), while the parameter “*a*” (green–red) was modeled as Δ*a* (increase of red tone or browning index).

The data on the microbiological counts, pH, and color were analyzed by means of one-way or multi-factorial analysis of variance (ANOVA) and Tukey’s test as the *post hoc* comparison test (*P* < 0.05), while sensory scores were treated through Kruskal–Wallis test (*P* < 0.05). Statistic was done through the software Statistica for Windows ver. 12.0 (Statsoft, Tulsa, OK).

## Results

The results for the first step (effect of different dipping times) are shown in Table 2. Immediately after the immobilization, the viable count of all strains on apple pieces was > 7 log cfu/g for all contact times (15–30 min); it did not experience a significant decrease throughout time (*P* > 0.05).

**TABLE 2:** Microbial cell load (log cfu/g) of *Limosilactobacillus reuteri* DSM 20016, *Bifidobacterium animalis* subsp. *lactis* DSM 10140, and *Lactiplantibacillus plantarum* c16 and c19 immobilized for 15, 20, 25, and 30 min on apple pieces and stored at 4°C for 12 days.

**Microorganisms**	**Days**	**Log cfu/g**			
		**15**	**20**	**25**	**30**
*L. reuteri* DSM 20016	0	7.61 ± 0.02	7.60 ± 0.08	7.60 ± 0.07	7.38 ± 0.02
	2	7.70 ± 0.05	8.00 ± 0.06	8.08 ± 0.12	8.08 ± 0.12
	7	7.56 ± 0.50	7.26 ± 0.01	7.25 ± 0.07	7.64 ± 0.54
	12	7.72 ± 0.02	7.20 ± 0.01	7.98 ± 0.08	7.96 ± 0.24
		ns*	ns	ns	ns
*Bifidobacterium animalis* subsp. *lactis* DSM 10140	0	8.07 ± 0.06	8.20 ± 0.14	8.40 ± 0.00	8.19 ± 0.10
	2	7.96 ± 0.08	7.54 ± 0.61	7.61 ± 0.20	7.12 ± 0.16
	7	8.33 ± 0.03	8.30 ± 0.01	8.18 ± 0.23	8.14 ± 0.06
	12	8.33 ± 0.03	8.30 ± 0.01	8.00 ± 0.00	8.06 ± 0.08
		Ns	ns	ns	ns
*L. plantarum* c16	0	7.98 ± 0.31	8.12 ± 0.25	8.00 ± 0.23	7.88 ± 0.17
	2	8.36 ± 0.34	8.08 ± 0.16	8.33 ± 0.04	8.59 ± 0.01
	7	8.35 ± 0.06	8.32 ± 0.03	8.32 ± 0.03	8.27 ± 0.05
	12	8.16 ± 0.02	8.22 ± 0.03	8.26 ± 0.00	8.33 ± 0.04
		Ns	ns	ns	ns
*L. plantarum* c16	0	8.45 ± 0.42	8.43 ± 0.15	8.42 ± 0.07	8.33 ± 0.09
	2	8.12 ± 0.17	8.26 ± 0.18	8.34 ± 0.08	8.43 ± 0.38
	7	8.36 ± 0.08	8.35 ± 0.01	8.38 ± 0.01	8.43 ± 0.04
	12	8.10 ± 0.01	8.20 ± 0.04	8.33 ± 0.04	8.30 ± 0.01
		Ns	ns	ns	ns

Mean ± standard deviation. *ns, the data for each contact time were analyzed by one-way ANOVA and Tukey’s test and the differences were not significant (P > 0.05).

For the second step, this research focused only on *Bifidobacterium animalis* subsp. *lactis* DSM 10140 due to its interesting functional properties. Therefore, the viability of *B. animalis* subsp. *lactis* 10140 on apple pieces coated with sodium alginate and gelatin and stored at 4 and 8°C for 10 days was evaluated.

The contact time was set to 15 min. pH and color measurements and sensory assessment were also performed.

The results obtained with *B. animalis* DSM 10140 were modeled as Δ*C* (change in the viable count) and analyzed through MANOVA, to point out the effects of time and coating (kind of sample and storage temperature). Coating and time affected Δ*C* as individual terms as well as in interaction with temperature, although coating was the most significant factor (Table 3).

**TABLE 3:** Statistical effects of coating, storage time, and temperature on Δ*C* value (increase/decrease of viable count) for *Bifidobacterium animalis* subsp. *lactis* DSM 10140. Multi-factorial ANOVA and Tukey’s test (*P* < 0.05).

**Variables**	**SS**	**df**	***F***
Time	0.44	4	3.66
Temperature	–*	–	–
Coating	0.78	2	12.86
Time*temperature	–	–	–
Time*coating	–	–	–
Temperature*coating	–	–	–
Time*temperature*coating	0.83	8	3.42

SS, sum of squares. df, degrees of freedom; F, Fisher test. *Not significant.

**FIGURE 1:**
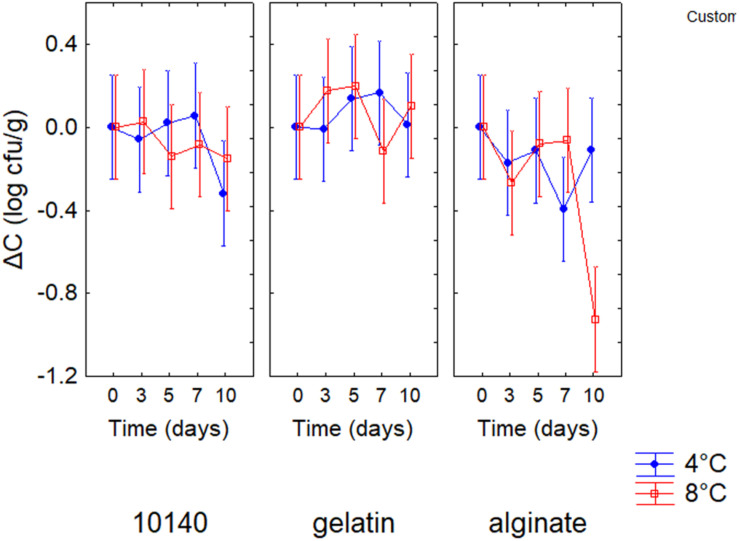
Decomposition of the statistical hypothesis for the interaction storage time/temperature/coating on Δ*C* (increase/decrease of viable count) for *Bifidobacterium animalis* subsp. *lactis* DSM 10140. Bars denote 95% confidence intervals. 10140, apple + *B. animalis* DSM 10140; gelatin, apple + *B. animalis* DSM 10140 + gelatin; alginate, apple + *B. animalis* DSM 10140 + sodium alginate.

The evaluation of the standardized effects does not provide information on “how much” an independent factor (or categorical predictor) affects the dependent variable. A quantitative estimation is possible through the decomposition of the statistical hypotheses. The decomposition for Δ*C* is shown in Figure 1; positive values of Δ*C* indicate an increase of probiotic count, while negative values mean a reduction of *B. animalis* DSM 10140 count throughout time. Generally, gelatin coating did not exert a negative effect on the viability of *B. animalis* DSM 10140; on the other hand, alginate coating caused a significant decrease of probiotic count at 8°C after 10 days of storage (Δ*C*, -1.2 log cfu/g).

**FIGURE 2:**
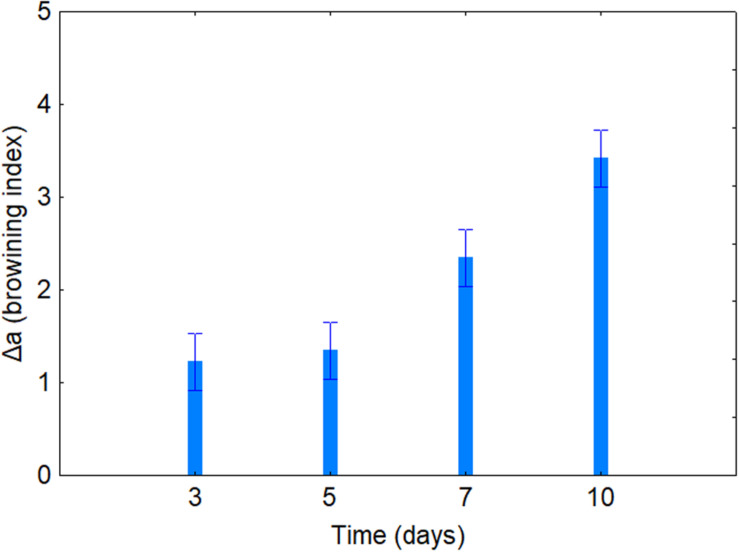
Decomposition of the statistical hypothesis for the individual effects of time on Δ*a* value (browning index). Bars denote 95% confidence intervals.

**FIGURE 3:**
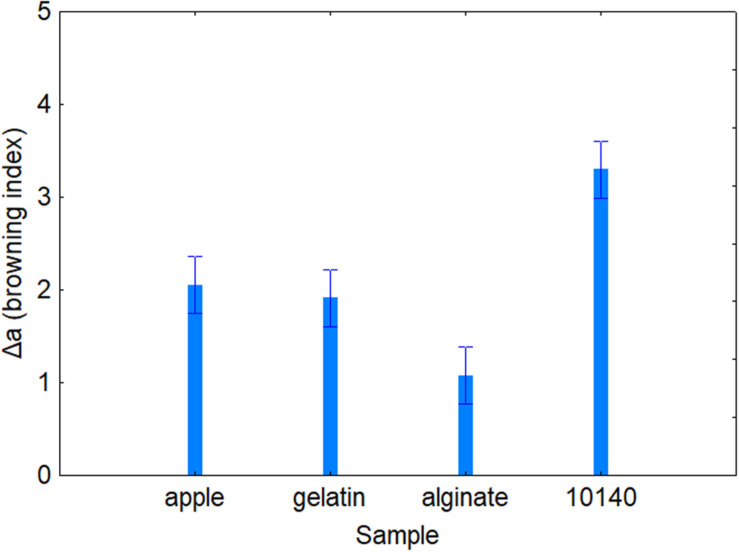
Decomposition of the statistical hypothesis for the individual effects of coating on Δ*a* value (browning index). Bars denote 95% confidence intervals. Apple, control; 10140, apple + *Bifidobacterium animalis* subsp. *lactis* DSM 10140; gelatin, apple + *B. animalis* DSM 10140 + gelatin; alginate, apple + *B. animalis* DSM 10140 + sodium alginate.

**FIGURE 4:**
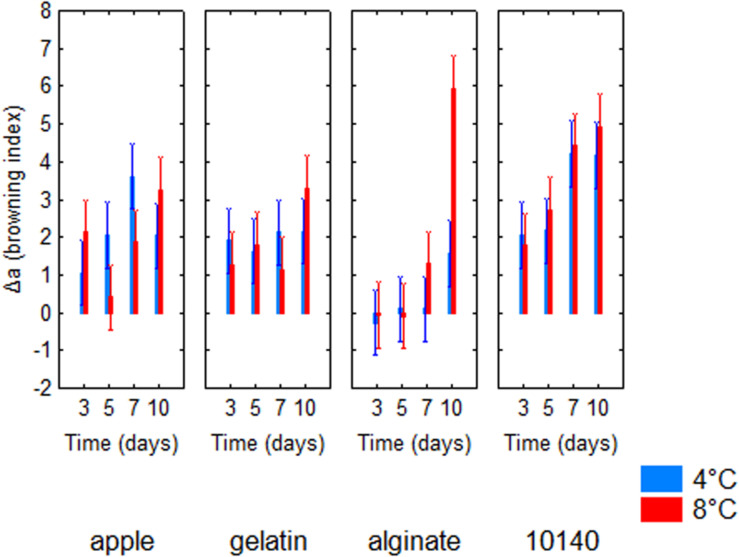
Decomposition of the statistical hypothesis for the interaction time/temperature/coating on Δ*a* value (browning index). Bars denote 95% confidence intervals. Apple, control; 10140, apple + *Bifidobacterium animalis* subsp. *lactis* DSM 10140; gelatin, apple + *B. animalis* DSM 10140 + gelatin; alginate, apple + *B. animalis* DSM 10140 + sodium alginate.

Table 4 shows the standardized effects for the browning index (Δ*a*); time, temperature, and coating were statistically significant both as individual and interactive terms. In particular, time played a major role (*F* = 43.14), followed by coating (*F* = 34.90) and finally by temperature (*F* = 4.64). As expected, browning index increased throughout time (Figure 2); the decomposition of the statistical hypothesis for coating pointed out the different effects of probiotic and coating on browning index. The probiotic caused a significant increase of Δ*a*; however, coating counteracted this effect (Figure 3). Finally, the worst effect on color (highest increase of Δ*a*) was found in alginate-coated pieces, stored at 8°C (Figure 4).

**TABLE 4:** Statistical effects of coating, storage time and temperature on Δ*a* value (browning index) of *Bifidobacterium animalis* subsp. *lactis* 10140. Multi-factorial ANOVA and Tukey’s test (*P* < 0.05).

**Variables**	**SS**	**df**	***F***
Time	125.08	3	43.14
Temperature	4.49	1	4.64
Coating	101.20	3	34.90
Time*temperature	32.35	3	11.16
Time*coating	53.80	9	6.19
Temperature*coating	16.71	3	5.76
Time*temperature*coating	28.10	9	3.23

*SS, sum of squares. df, degrees of freedom; F, Fisher test.*

The pH of apples was 2.8–2.9; alginate and gelatin coating, as well as the inoculation of microorganisms, did not affect it, probably due to the low temperature. pH, in fact, was at 2.68–2.76 throughout storage (data not shown).

**FIGURE 5:**
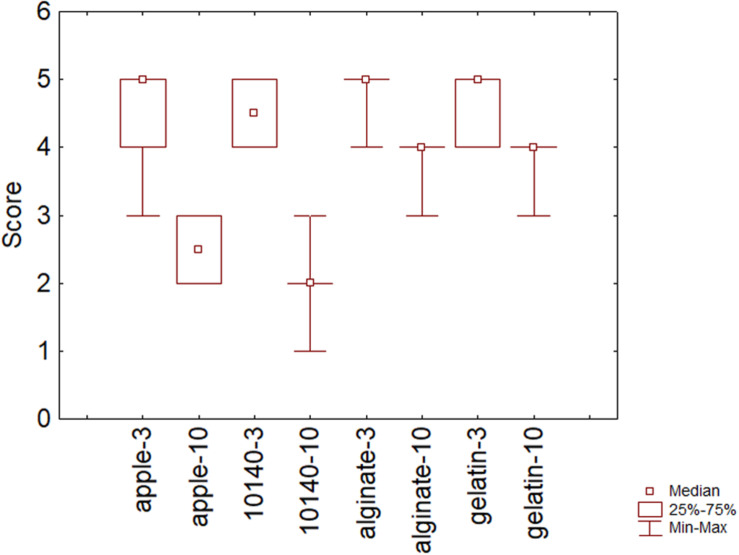
Sensory scores on the overall acceptability after 3 and 10 days of storage at 4°C. Apple, control; 10140, apple + *Bifidobacterium animalis* subsp. *lactis* DSM 10140; gelatin, apple + *B. animalis* DSM 10140 + gelatin; alginate, apple + *B. animalis* DSM 10140 + sodium alginate. 3 and 10, 3 and 10 days of storage.

Sensory scores were also assessed; Figure 5 shows the overall quality after 3 and 10 days of storage at 4°C. As expected, scores decreased throughout time (*P* = 0.02) mainly in apple without coating (apple) and in apple chips with *B. animalis* DSM 10140 and without coating (10140). Some panelists regarded as inacceptable the sample containing only the probiotic after 10 days with a score of 1.

The use of coating (alginate and gelatin) could counteract this phenomenon; in fact, the samples with *B. animalis* DSM 10140 and coating retained a higher sensory score after 10 days.

## Discussion

Although fermented dairy products are the most common foods through which humans take probiotics, many non-dairy or non-traditional probiotic products are now marketed in many countries (Ranadheera et al., 2017). Fruit and vegetables are rich in nutrients and, when combined with probiotics, can positively affect human health; therefore, using fruits as carriers for probiotics is highly advantageous and is widely accepted by consumers (Martins et al., 2013).

Moreover, fruits are good substrates for probiotics since they have nutrients and morphological structures favoring microbial growth (Martins et al., 2013). In addition, compared to dairy products, fruits are free of cholesterol, lactose, and allergens, and this is another reason for the emerging trend toward the consumption of non-dairy foods.

Some authors proposed fruit chips as carriers for lactobacilli and yeasts (among others, Speranza et al., 2018; Altieri et al., 2019), but few evidences can be found on bifidobacteria (Rahmdel et al., 2019). Therefore, considering the potential applications of these types of products in the food industry, the feasibility of developing a protocol for the production of apple pieces as probiotic carriers with two different coatings was investigated.

The first goal of this research was to study the effect of contact time on strain adsorption on apple pieces. The immobilization of probiotics on fruit pieces is generally the result of a natural deposition of microorganisms on the surface of fruit and vegetable chips (Kourkoutas et al., 2005, 2006; Gallo et al., 2014; Russo et al., 2014, 2015; Speranza et al., 2018). The yield of strain deposition could be affected by several variables, like the shape of piece, the duration of dipping in probiotic solution, the use of static conditions or agitation, etc. Focusing on the duration of the treatment, Russo et al. (2014, 2015) used a dipping time of 2 min for bananas and cantaloupe melon, while other authors (for example, Kourkoutas et al., 2005, 2006) used a dipping time of 15 min. Preliminary results (Campaniello, unpublished results) showed that dipping for a few minutes did not assure a high deposition of probiotics, and the viable count after the process was 5–6 log cfu/g, i.e., lower than the break point for probiotics foods. Based on these results, a dipping time of at least 15 min was used; however, the results showed that the level of probiotics on apple pieces was not influenced by the contact time (from 15 to 30 min); thus, for the easiness of the process (a technological innovation should be feasible without affecting productivity and operational capacity), a contact time of 15 min (the lowest one) was regarded as acceptable for the second step of this research.

A second requisite to design probiotic food is the viability of probiotics throughout storage (technological robustness of the microbial targets); apple chips are not a “friendly” environment due to the low pH; thus, strain viability was assessed after the evaluation of the effect of contact time. Results show that all strains survived on apple pieces throughout the storage at 4°C. These data are in line with some literature findings.

Rößle et al. (2010) loaded *Lacticaseibacillus rhamnosus* GG to fresh-cut apple wedges (cv. Braeburn) and found that the initial 10^8^ cfu/g inoculum remained stable throughout 10 days of storage at 2–4°C. Rahmdel et al. (2019) used apple pieces as carriers for *Bifidobacterium animalis* subsp. *lactis* BB-12; the count was higher than the threshold (6 log cfu/g) for the whole shelf life.

For the design of coated apple chips, only *B. animalis* subsp. *lactis* DSM 10140 was used because of its technological robustness (Speranza et al., 2012; Bevilacqua et al., 2013). However, the design of a probiotic food is not a simple inoculation of microorganism and a mere evaluation of viability throughout time. In fact, it is well known that due to their active metabolism, some probiotics continue to produce lactic acid or other metabolites, thus causing negative changes on organoleptic and sensory scores (Bevilacqua et al., 2016; Racioppo et al., 2017). A negative effect of probiotics in fruit chips was found by Speranza et al. (2018) with regard to *L. plantarum* c19 inoculated on apple pieces.

In addition, fruits and vegetables, when processed, are more perishable than the raw material; in fact, when fruits are minimally processed, natural barriers are removed through peeling and subjected to washing, cutting, and slicing. All these treatments cause tissue injuries and damage the integrity of the fruits, and the product becomes vulnerable to contamination, enzymatic oxidations, and structural alterations.

The negative effect of probiotics was also found in this research, as suggested by the increase of the browning index (more than in the control chips) as well as by a significant decrease of the overall quality perceived by the panelist. These effects could be the result of the pectolytic activity of *Bifidobacterium* spp. (Slováková et al., 2002; Flint et al., 2008) as well as of glucose/fructose metabolism. Although to some extent a partial degradation of pectin could be of interest, because bifidobacteria produce SCFA (short chain fatty acids) from some fibers, the reduction of sensory scores could be a problem.

The effects related to probiotic activity and perishable nature of minimally processed fruits can be prevented and/or counteracted through the use of artificial barriers surrounding the product as edible and coating films (Parreidt et al., 2018). In fact, edible films acting as selective barriers to moisture transfer, to oxygen uptake, to lipid oxidation, and to the loss of flavors and aromas are capable of improving the food quality (Tapia et al., 2007).

Due to its colloidal properties and ability to make strong gels or insoluble polymers, alginate is considered suitable for edible films and food coatings. Furthermore, it is classified as GRAS (generally regarded as safe) by the U.S. Food and Drug Administration (FDA), and the European Commission (EC) listed alginic acid and its salts (E400–E404) as an authorized food additive (Parreidt et al., 2018). Another possibility for edible coating is gelatin (Aguilar-Méndez et al., 2008; Gol and Ramana Rao, 2013), because of its barrier properties against oxygen and carbon dioxide (Jiang et al., 2010). Moreover, gelatin is a widespread ingredient in many formulations, and it is not perceived as a non-natural compound.

The use of alginate and gelatin coating counteracted the negative effects of both minimally processed fruits (perishable products) and probiotic (browning of apple chips) and assured the retention of sensory scores for a longer time. The performances of the two coatings were similar, although *B. animalis* experienced a 1-log viability loss on apple pieces with alginate stored at 8°C. There are no evidences to give an insight into this phenomenon, and further investigations are required.

Other researchers stressed the feasibility of coated fruit pieces with probiotics. Tapia et al. (2007) proposed alginate- and gellan-based edible films for apple and papaya chips; they found that coating was able to prevent the viability loss of *B. lactis* BB-12 on fresh-cut apple and papaya.

Speranza et al. (2018) conducted a study on apple (and melon) pieces as carriers for *L. plantarum.* Alginate and chitosan were used as coatings, but the results of this study demonstrated that alginate coating provided better performances while preserving the vitality of the probiotic strain.

Mannucci et al. (2017) used gelatin as edible coating on Fuji apple to evaluate the aroma profile, and the authors observed a reduction in the acetate esters (which generally increase with maturity) that induced a delay in ripening, as an effect of the presence of gelatin coating.

The results of this paper suggest the possibility of an effective scaling up at the industrial level of apple chips with *B. animalis* subsp. *lactis* DSM 10140 and coated with gelatin or alginate for a better retention of sensory scores; however, there are some open questions that should be addressed. First, bifidobacteria produce SCFA from pectins; thus, it could be of interest to assess if these compounds could be produced *in situ* as a way to enrich apple pieces with bioactive compounds. In addition, some other parameters should be assessed in the light of nutritional characterization of coated apple pieces (such as vitamins, sugar, etc.) to clarify the role of probiotics on the nutritive traits.

This paper addresses the technological challenge related to the design of a new carrier for *B. animalis* subsp. *lactis* DSM 10140; however, a future perspective of this research could be a focus on the functional effect of this product (viability of the probiotic throughout the transit into the gut, ability to induce SCFA production in the large intestine, and effect on the gut microbiota).

## Conclusion

This paper reports on the design of apple-based carriers for *B. animalis* subsp. *lactis* DSM 10140. The probiotic survived throughout storage at 4 and 8°C, but it could cause a worsening of color with an increase of browning index. However, the use of a coating (alginate- or gelatin-based) counteracted this effect.

Concerning the effect of the viability, gelatin coating showed a better performance than alginate.

The formulation proposed in this research could meet the approval of a large number of consumers; in fact, due to their convenience, fresh-cut fruits are considered very attractive. Then, if these ready-to-use foods are also healthy thanks to the presence of probiotic strains, their attractiveness further increases. Moreover, the gelatin coating could make the final product suitable for different types of use (e.g., pastry).

Further studies are necessary to evaluate, for example, the role of the probiotic, carried by vegetable foods, in the intestine, as well as the metabolites produced throughout storage and other nutritional traits.

## Data Availability Statement

The raw data supporting the conclusions of this article will be made available by the authors, without undue reservation, to any qualified researcher.

## Author Contributions

DC, MS, and MC: conceptualization. AB: software. BS and DC: investigation. MS and MC: resources. AB and DC: methodology, data curation, and writing – original draft preparation. MC: supervision and funding acquisition. DC, AB, BS, MS, and MC: writing – review and editing. All authors have read and agreed to the published version of the manuscript.

## Conflict of Interest

The authors declare that the research was conducted in the absence of any commercial or financial relationships that could be construed as a potential conflict of interest.

## References

[B1] Aguilar-Méndez M. A. San Martín-Martínez E. Tomás S. A. Cruz-Orea A. Jaime-Fonseca M. R. (2008). Gelatine-starch films: physicochemical properties and their application in extending the post-harvest shelf life of avocado (*Persea americana*). *J. Sci. Food Agric.* 88 185–193. 10.1002/jsfa.3068

[B2] Altieri C. Campaniello D. Speranza B. Sinigaglia M. Corbo M. R. Bevilacqua A. (2019). Immobilization of *Saccharomyces cerevisiae* on apple pieces to produce cider. *Fermentation* 5:74 10.3390/fermentation5030074

[B3] Bevilacqua A. Altieri C. Corbo M. R. Sinigaglia M. Ouoba L. I. I. (2010). Characterization of lactic acid bacteria isolated from italian bella di cerignola table olives: selection of potential multifunctional starter cultures. *J. Food Sci.* 75 M536–M544. 21535510 10.1111/j.1750-3841.2010.01793.x

[B4] Bevilacqua A. Campaniello D. Corbo M. R. Maddalena L. Sinigaglia M. (2013). Suitability of *Bifidobacterium* spp. and *Lactobacillus plantarum* as probiotics intended for fruit juices containing citrus extracts. *J. Food Sci.* 78 1764–1771. 10.1111/1750-3841.12280 24245895

[B5] Bevilacqua A. Casanova F. P. Petruzzi L. Sinigaglia M. Corbo M. R. (2016). Using physical approaches for the attenuation of lactic acid bacteria in an organic rice beverage. *Food Microbiol.* 53 1–8. 10.1016/j.fm.2015.08.005 26678123

[B6] Flint H. J. Bayer E. A. Rincon M. T. Lamed R. White B. A. (2008). Polysaccharide utilization by gut bacteria: potential for new insights from genomic analysis. *Nat. Rev. Microbiol.* 6 121–131. 10.1038/nrmicro1817 18180751

[B7] Gallo M. Bevilacqua A. Speranza B. Sinigaglia M. Corbo M. R. (2014). Alginate beads and apple pieces as carriers for *Saccharomyces cerevisiae* var. *boulardii*, as representative of yeast functional starter cultures. *Int. J. Food Sci. Technol.* 49 2092–2100. 10.1111/ijfs.12518

[B8] Gol N. B. Ramana Rao T. V. (2013). Influence of zein and gelatin coatings on the postharvest quality and shelf life extension of mango (*Mangifera indica* L.). *Fruits* 69 101–115. 10.1051/fruits/2014002

[B9] Jiang M. Liu S. Du X. Wang Y. (2010). Physical properties and internal micro-structures of films made from catfish skin gelatin and triacetin mixtures. *Food Hydrocoll.* 24 105–110. 10.1016/j.foodhyd.2009.08.011

[B10] Kandylis P. Mantzari A. Koutinas A. A. Kookos I. K. (2012). Modelling of low temperature wine-making, using immobilized cells. *Food Chem.* 133 1341–1348. 10.1016/j.foodchem.2012.02.022

[B11] Karel S. F. Libicki S. B. Robertson C. R. (1985). The immobilization of whole cells: engineering principle. *Chem. Eng. Sci.* 40 1321–1354. 10.1016/0009-2509(85)80074-9

[B12] Kopsahelis N. Panas P. Kourkoutas Y. Koutinas A. A. (2007). Evaluation of the thermally dried immobilized cells of *Lactobacillus delbrueckii* subsp. *bulgaricus* on apple pieces as a potent starter culture. *J. Agric. Food Chem.* 55 9829–9836. 10.1021/jf0719712 17985843

[B13] Kourkoutas Y. Kanellaki M. Koutinas A. A. (2006). Apple pieces as immobilization support of various microorganisms. *LWT Food Sci. Technol.* 39 980–986. 10.1016/j.lwt.2006.02.024

[B14] Kourkoutas Y. Xolias V. Kallis M. Bezirtzoglou E. Kanellaki M. (2005). Lactobacillus casei cell immobilization on fruit pieces for probiotic additive, fermented milk and lactic acid production. *Process Biochem.* 40 411–416. 10.1016/j.procbio.2004.01.029

[B15] Mannucci A. Serra A. Remorini D. Castagna A. Mele M. Scartazza A. (2017). Aroma profile of Fuji apples treated with gelatin edible coating during their storage. *LWT Food Sci. Techol.* 85 28–36. 10.1016/j.lwt.2017.06.061

[B16] Martins E. M. F. Ramos A. M. Vanzela E. S. L. Stringheta P. C. Pinto C. L. O. Martins J. M. (2013). Products of vegetable origin: a new alternative for the consumption of probiotic bacteria. *Food Res. Int.* 51 764–770. 10.1016/j.foodres.2013.01.047

[B17] Mitropoulou G. Nedovic V. Goyal A. Kourkoutas Y. (2013). Immobilization technologies in probiotic food production. *J. Nutr. Metab.* 2013 1–15. 10.1155/2013/716861 24288597 PMC3830840

[B18] Moreno-García J. García-Martínez T. Mauricio J. C. Moreno J. (2018). Yeast immobilization systems for alcoholic wine fermentations: actual trends and future perspectives. *Front. Microbiol.* 9:241. 10.3389/fmicb.2018.00241 29497415 PMC5819314

[B19] Parreidt T. S. Müller K. Schmid M. (2018). Alginate-based edible films and coatings for food packaging applications. *Foods* 7:170 10.3390/foods7100170 PMC6211027 30336642

[B20] Poreda A. Tuszyñski T. Zdaniewicz M. Sroka P. Jakubowski M. (2013). Support materials for yeast immobilization affect the concentration of metal ions in the fermentation medium. *J. Inst. Brew.* 119 164–171.

[B21] Racioppo A. Corbo M. R. Piccoli C. Sinigaglia M. Speranza B. Bevilacqua A. (2017). Ultrasound attenuation of lactobacilli and bifidobacteria: effect on some technological and probiotic properties. *Int. J. Food Microbiol.* 21 243–278. 10.1016/j.ijfoodmicro.2016.12.011 28038333

[B22] Rahmdel S. Jahed-Khaniki G. Abdollahzadeh S. M. Shekarforoush S. S. Mazloomi S. M. (2019). Development of fresh-cut apple slices enriched with probiotic strain *Bifidobacterium animalis* subsp. *lactis BB-*12. *Int. J. Probiot. Prebiot.* 14 37–44. 10.37290/ijpp2641-7197.14:37-44

[B23] Ranadheera C. S. Vidanarachchi J. K. Rocha R. S. Cruz A. G. Ajlouni S. (2017). Probiotic delivery through fermentation: dairy vs. non-dairy beverages. *Fermentation* 3:67 10.3390/fermentation3040067

[B24] Rößle C. Auty M. A. E. Brunton N. Gormley R. T. Butler F. (2010). Evaluation of fresh-cut apple slices enriched with probiotic bacteria. *Innov. Food Sci. Emerg. Technol.* 11 203–209. 10.1016/j.ifset.2009.08.016

[B25] Russo P. De Chiara M. L. V. Vernile A. Amodio M. L. Arena M. P. Capozzi V. (2014). Fresh-cut pineapple as a new carrier of probiotic lactic acid bacteria. *Biomed. Res. Int.* 2014:309183. 10.1155/2014/309183 PMC4100397 25093163

[B26] Russo P. Peña N. de Chiara M. L. V. Amodio M. L. Colelli G. Spano G. (2015). Probiotic lactic acid bacteria for the production of multifunctional fresh-cut cantaloupe. *Food Res. Int.* 77 762–772. 10.1016/j.foodres.2015.08.033

[B27] Slováková L. Dušková D. Marounek M. (2002). 1.1 Fermentation of pectin and glucose, and activity of pectin-degrading enzymes in the rabbit caecal bacterium *Bifidobacterium pseudolongum*. *Lett. Appl. Microbiol.* 35 126–130. 10.1046/j.1472-765x.2002.01159.x 12100587

[B28] Speranza B. Bevilacqua A. Sinigaglia M. Corbo M. R. (2012). Shelf life definition for Italian anchovies inoculated with *Lactobacillus plantarum* and *Bifidobacterium animalis* subsp. *lactis*. *Innov. Food Sci. Emerg. Technol.* 16 171–180. 10.1016/j.ifset.2012.05.009

[B29] Speranza B. Campaniello D. Bevilacqua A. Altieri C. Sinigaglia M. Corbo M. R. (2018). Viability of *Lactobacillus plantarum* on fresh-cut chitosan and alginate-coated apple and melon pieces. *Front. Microbiol.* 9:2538 10.3389/fmicb.2018.2538 PMC6205977 30405587

[B30] Tapia M. S. Rojas-Grau M. A. Rodríguez E. J. Ramírez J. Carmona A. Martin-Belloso O. (2007). Alginate- and gellan-based edible films for probiotic coatings on fresh-cut fruits. *J. Food Sci.* 72 E190–E196. 17995771 10.1111/j.1750-3841.2007.00318.x

[B31] Thakur M. Satwadhar P. N. Deshpande H. W. (2015). Exploiting fruits and vegetables for development of probiotic products. *Trends Biosci.* 8 6039–6046.

[B32] Valero-Cases E. Cerdá-Bernad D. Pastor J. J. Frutos M. J. (2020). Non-dairy fermented beverages as potential carriers to ensure probiotics, prebiotics, and bioactive compounds arrival to the gut and their health benefits. *Nutrients* 12:1666. 10.3390/nu12061666 PMC7352914 32503276

